# “To live a meaningful life, be yourself and make yourself”: Haoyan Jen—a pioneer of China’s environmental microbiology

**DOI:** 10.1007/s13238-020-00781-z

**Published:** 2020-09-04

**Authors:** Xin Zhang, Huan Liu

**Affiliations:** 1 Institute of Biophysics, Chinese Academy of Sciences, Beijing, 100101 China; 2 Wuhan Institute of Virology, Chinese Academy of Sciences, Wuhan, 430071 China; 3 Chinese Center for Disease Control and Prevention, Beijing, 102206 China; 4 State Key Laboratory of Virology, Wuhan, 430072 China

There is a man who improves our living and working environment by turning pesticide residues in the field into nutrients for the crops, turning such wastes as paper and sawdust into sugar, which is then converted into liquid alcohol fuel, or purifying malodorous and toxic industrial wastewater into clean and non-toxic water. He is Prof. Haoyan Jen (简浩然), a pioneer of China’s environmental microbiology.

Prof. Haoyan Jen, a famous microbiologist, is also one of the founders of environmental microbiology in China. He is a member of the Chinese Society for Environmental Sciences, Chinese Society for Microbiology, Chinese Society of Biotechnology, and New York Academy of Sciences, as well as a special allowance expert of the State Council of China. He devoted himself to microbiology and environmental biology, to which he made major contributions such as the successful removal of phenol from industrial wastewater with biochemical methods for the first time in China, and obtaining two highly-effective phenol degrading bacterial strains, which were used in a collaboration on science and technology between China and five Eastern European countries. He is the author of inventions-creations certified by the State Scientific and Technological Commission for his “Microbial Degumming of Cotton Bark and Wild Fibers”; development of a special material to prevent microbiologically influenced corrosion of underground pipelines in China, a study on microbial plasmid-assisted molecular breeding for the degradation of persistent pollutants to control environmental pollution, and the development of a BOD microbial sensor consisting of immobilized bacterial cells (*Pseudomonas* sp. A_4_, isolated from active sludge). He was awarded the Second Prize of Science and Technology Progress and the Second Prize of Natural Sciences by the Chinese Academy of Sciences for his “Application of Microorganisms in the Treatment of Industrial Pollution Sources”, supported by the Key Science and Technology Project of the National “Seventh Five-Year Plan” (Han, 1993).

Prof. Jen was a native of Nanhai, Guangdong Province, born in Hong Kong, China on December 25, 1911. In 1937, he received his Master’s Degree from Sun Yat-sen University, and became the first graduate in soil microbiology in China. In 1946, he pursued further studies at the University of Wisconsin in the United States. Two years later, he received his PhD in soil microbiology. Appreciating his excellent academic record, his tutor recommended him to join the American Science Society and suggested him to work in the United States. However, he returned to his motherland on the eve of the founding of the People’s Republic of China in 1949 with a passion to serve the country. Then, he worked at the Chinese Academy of Sciences and participated in the establishment of the Wuhan Institute of Microbiology (now Wuhan Institute of Virology).

In 1954, vast amounts of wastewater containing phenol were being dumped into farmlands and ponds by a timber preservation plant, leading to pollution and death of crops and fish, which caused great loss for the farmers. The government made an immediate response to the pollution. Considering the limited economic conditions in China, Prof. Jen, who was assigned to take charge of this work, decided to adopt the highly-effective and cost-saving biological approach to the treatment of wastewater pollution. He isolated two strains of highly-efficient phenol-degrading bacteria from the contaminated soil near the plant, cultivated them and applied large amounts to the wastewater. The magical bacteria “ate up” the harmful phenol. Prof. Jen purified wastewater containing phenol with biological methods in China for the first time, which not only opened a new chapter in the development of environmental science in China, but also assisted five Eastern European countries in effectively treating pollution of wastewater containing phenol.

In 1980, there were 6.6 million hectares of farmland contaminated with pesticides across China. To address the increasing damage to the ecological environment, Prof. Jen led the research on “Microbial Degradation and Application of Chemical Pesticides”, in which he successfully selected new bacterial strains capable of degrading organochlorine pesticides, organophosphorus pesticides, sulfur phosphorus and polychlorinated compounds, and discovered BHC degrading bacteria and plasmids. In his degradation experiments with two isolated fungal strains and one yeast strain, a significant reduction of β-BHC was found in the culture medium, with a removal rate of over 90 percent (Wang et al., 1986). During the period, he succeeded in the molecular breeding of biodegrading bacteria in China for the first time by means of then-novel plasmid-assisted genetic engineering. The natural evolution process was far from being able to meet the urgent needs in response to environmental pollution, while plasmid-assisted molecular breeding offered a promising method for protecting the environment.

In 1985, Prof. Jen, in his 70s, felt heartbroken by the severely damaged natural environment, when he saw a large amount of colored toxic wastewater released from a printing and dyeing factory into the river on his way to visit family and relatives. He stayed at home for a very short period of time and returned to the Institute. At that time, someone had persuaded him not to waste time on such research with few economic benefits, while Prof. Jen insisted on carrying out the project “Application of Microorganisms in the Treatment of Industrial Pollution Sources”. After more than 1,000 days and nights, the project was successfully completed. He adopted several measures to treat textile wastewater, including decolorizing bacteria immobilized onto porous ceramics, which was highly effective and had a long-term effect. Compared with conventional treatment, it has advantages such as short decolorization time, desirable effect, resistance against bacterial contamination and continuous use (Huang et al., 1990). He was also successful in the isolation of the symbiotic bacteria SB_1_ to degrade PVA (Wang et al., 1991). The artificially bred microbes could purify 50,000 cubic meters of wastewater per day. Prof. Jen was awarded the Second Prize for Science and Technology Progress by the Chinese Academy of Sciences for this project in 1991. At the Acceptance Conference of the Key Science and Technology Project of the “Seventh Five-Year Plan”, he first put forward “biological treatment for the environment”, which was 400 days earlier than it was proposed internationally.

A microbial sensor consisting of living microorganism immobilized on the surface of an electrode is a sensitive material for molecular identification. It offers advantages of rapid, simple and repeatable operation. Considering the limited life span and application range of microbial sensors at that time, Prof. Jen, together with two scientists, isolated *Pseudomonas* sp. A_4_ from active sludge and developed a microbial BOD sensor in 1986. Using this sensor, the BOD of a sample solution could be measured within 15 min, while offering good correlation with traditional methods, and a freshly prepared sensor could be used over 6 weeks (Zhang et al., 1986).

**Figure 1:**
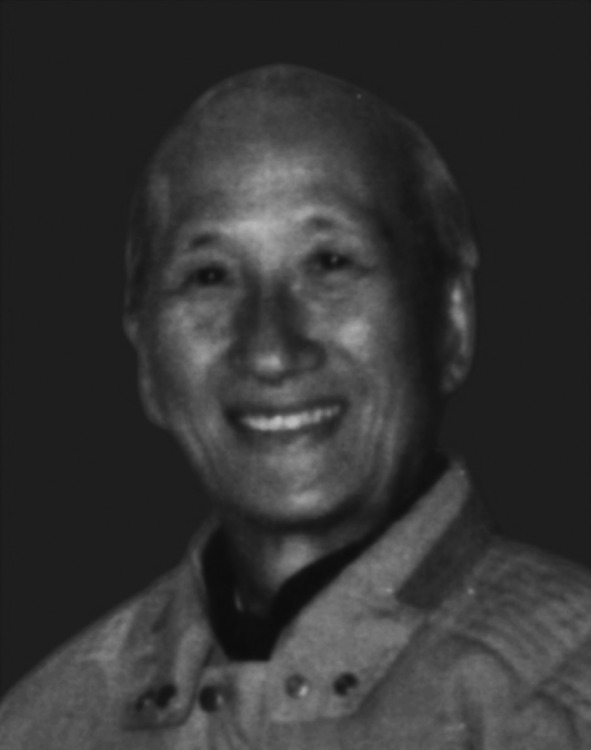
Prof. Haoyan Jen

**Figure 2:**
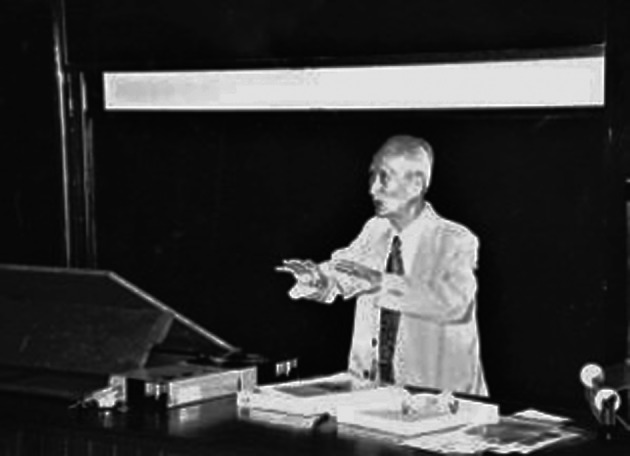
Prof. Haoyan Jen in class

For more than 70 years, Prof. Jen devoted himself to science and benefiting humanity by reducing pollution, eliminating toxicity, and turning trash into treasure. He advocated that to do well in scientific research, one must have three “tools”, including professional skills, language skills, and practical skills (Figs. 1 and 2). He also believed that mathematics can open another door for us by broadening the scientific horizon (Qing, 2012). Over several decades, Prof. Jen cultivated many scientific and technological talents in China. He was open-minded and modest, and continued working at the forefront of scientific research at nearly 90 years old, even overtime or in illness. “To live a meaningful life, be yourself and make yourself”, Prof. Jen left inexhaustible spiritual wealth for us with his firm belief in science and selfless dedication to the scientific cause, as well as his glorious hardworking leadership and benevolence, which will forever inspire people to strive for the revitalization of the nation and benefiting humanity.
